# Is Exposure to Macondo Oil Reflected in the Otolith Chemistry of Marsh-Resident Fish?

**DOI:** 10.1371/journal.pone.0162699

**Published:** 2016-09-28

**Authors:** Paola C. López-Duarte, F. Joel Fodrie, Olaf P. Jensen, Andrew Whitehead, Fernando Galvez, Benjamin Dubansky, Kenneth W. Able

**Affiliations:** 1 Rutgers University Marine Field Station, Department of Marine and Coastal Sciences, Rutgers, The State University of New Jersey, Tuckerton, New Jersey, United States of America; 2 Institute of Marine Sciences & Department of Marine Sciences, University of North Carolina at Chapel Hill, Morehead City, North Carolina, United States of America; 3 Department of Marine and Coastal Sciences, Rutgers, The State University of New Jersey, New Brunswick, New Jersey, United States of America; 4 Environmental Toxicology Department, University of California Davis, Davis, California, United States of America; 5 Department of Biological Sciences, Louisiana State University, Baton Rouge, Louisiana, United States of America; 6 Department of Biological Sciences, University of North Texas, Denton, Texas, United States of America; Department of Agriculture and Water Resources, AUSTRALIA

## Abstract

Genomic and physiological responses in Gulf killifish (*Fundulus grandis*) in the northern Gulf of Mexico have confirmed oil exposure of resident marsh fish following the Macondo blowout in 2010. Using these same fish, we evaluated otolith microchemistry as a method for assessing oil exposure history. Laser-ablation inductively-coupled-plasma mass spectrometry was used to analyze the chemical composition of sagittal otoliths to assess whether a trace metal signature could be detected in the otoliths of *F*. *grandis* collected from a Macondo-oil impacted site in 2010, post-spill relative to pre-spill, as well as versus fish from areas not impacted by the spill. We found no evidence of increased concentrations of two elements associated with oil contamination (nickel and vanadium) in *F*. *grandis* otoliths regardless of Macondo oil exposure history. One potential explanation for this is that Macondo oil is relatively depleted of those metals compared to other crude oils globally. During and after the spill, however, elevated levels of barium, lead, and to a lesser degree, copper were detected in killifish otoliths at the oil-impacted collection site in coastal Louisiana. This may reflect oil contact or other environmental perturbations that occurred concomitant with oiling. For example, increases in barium in otoliths from oil-exposed fish followed (temporally) freshwater diversions in Louisiana in 2010. This implicates (but does not conclusively demonstrate) freshwater diversions from the Mississippi River (with previously recorded higher concentrations of lead and copper), designed to halt the ingress of oil, as a mechanism for elevated elemental uptake in otoliths of Louisiana marsh fishes. These results highlight the potentially complex and indirect effects of the Macondo oil spill and human responses to it on Gulf of Mexico ecosystems, and emphasize the need to consider the multiple stressors acting simultaneously on inshore fish communities.

## Introduction

The Gulf of Mexico (GOM) is a highly dynamic and productive ecosystem that supports recreational and commercial fishing, tourism, as well as significant oil and gas exploration and extraction industries (~600 million barrels per year) [1,2]. On April 20, 2010, the explosion of the Deepwater Horizon drilling rig precipitated the 84-day release of an estimated 4.5 million barrels of Louisiana crude oil into the Gulf of Mexico [3]. Although the release of oil at great depth impacted deep sea ecosystems [4–6], surface oil also reached the coastline by May, 2010, and ultimately grounded along 25% of the beach and saltmarsh shorelines surveyed in Gulf states [7].

Louisiana coastal wetlands are inextricably linked to the health of GOM fisheries [8–10]. Therefore, there has been considerable interest in describing organismal [11–15] and population-level responses [16,17] of saltmarsh-associated nekton to oil pollution throughout the northern GOM. The Gulf killifish (*Fundulus grandis*) has served as a valuable model species to assess oil-spill impacts. *Fundulus* spp. are numerically dominant marsh residents [17], serve as an important trophic link in estuarine food webs [18], exhibit considerable site fidelity [19,20], and perform well in laboratory trials without demonstrating handling artifacts on fitness [21].

*Fundulus grandis* has been a focal species in post-spill impact assessments, but studies report mismatched outcomes. Negative impacts of oiling are consistently documented at the organismal level as revealed through genomic, physiological, and developmental responses [11–15], while population-level impacts have not been detected [16,17]. A recent review by Fodrie et al. [22] highlighted the value of testing for sublethal effects of oil toxicity on fishes such as *F*. *grandis* as a way of reconciling this apparent divergence in organismal and population-level studies. Sublethal effects could either result in lagged effects at the population level (thus not yet registering in population surveys), or perhaps affect vital rates that simply do not have strong ties to individual or population-level fitness. Six years post-spill, however, it is difficult to collect fish and know to what degree individuals encountered oil during 2010, and therefore what expectation should exist regarding the potential for oil-related sublethal effects among those fish (e.g., reduced growth).

One tool for potentially recovering the oil-exposure history of individual fish relies on examination of the chemical composition of otoliths. Otoliths, “ear stones” of calcium carbonate located in the inner ear of teleost fish, grow in daily increments around a central core. As the otolith grows, trace elements from the environment are continuously incorporated into successive rings in a manner that reflects the environmental conditions experienced by that individual [23]. For instance, salinity and temperature regimes experienced by fish are often reflected in the concentrations of strontium (Sr) and/or barium (Ba) [24–27].

Previous research in experimental mesocosms demonstrated that crude oil constituents, including magnesium (Mg), chromium (Cr), and Sr, were accumulated in juvenile flatfish–likely through both food intake and via exchange across the gills and then incorporated into the otoliths [28]. Nickel (Ni) and vanadium (V), also oil constituents [29], have been detected in otoliths and used as a fingerprint for the magnitude and duration of oil contact by invertebrates [30,31] and fishes [32]. Macondo oil has several constituents, including Mg, V, Mn, Fe, Co, Ni, Cu, Ba, and Pb, which occur at varying concentrations relative to oils from other sources [33,34]. Indeed, oil constituents vary significantly depending on the source of the oil [29], and therefore the suite of chemical markers used to identify oil contact may be unique to each spill (sensu [35]). Despite the promise of this approach, to date, there is only one published study that has evaluated otolith-based markers for detecting oil contact in GOM fishes following the Macondo spill [36]. In that study, *F*. *grandis* were collected from northern GOM coastal marshes in 2012–2013 at sites that were either oiled in 2010 by the Macondo Spill based on Shoreline Cleanup and Assessment Technique (SCAT) surveys [7] or near an active oil refinery. The results of that study indicate that there was no evidence of oil exposure in the otoliths of fish at oil-impacted sites 2–3 years after the spill.

To rigorously evaluate whether signatures of Macondo oil appear in the otoliths of fishes *in situ*, a positive control is needed, in which other independent markers have confirmed oil exposure for individual fishes. Throughout 2010, including before, during, and after the Macondo spill, Whitehead et al. [11] collected *F*. *grandis* from marsh systems in Louisiana, Mississippi, and Alabama in a Before-After-Control-Impact (BACI) design. At an oiled site, *F*. *grandis* exhibited a pattern of altered genome-wide gene expression in liver tissues that was indicative of exposure to oil. Within the gills of *F*. *grandis* exposed to oil, cytochrome CYP1A protein, a widely used biomarker of exposure to polycyclic aromatic hydrocarbons (PAH) [37–39], was elevated, correlating with the upregulation of associated genes coincident with the timing and arrival of oil to the northern GOM. These molecular responses were diagnostic of oil exposure [40] and were not consistent with other environmental variables such as salinity or temperature. Following this study, Dubansky et al. [13] evaluated multiple tissues from these fish from 2010 and additional sampling in 2011 from Louisiana. Again, genome-wide gene expression, this time in gills, and CYP1A protein in gill, liver, intestine, and kidney tissues were indicative of exposure to contaminating oil. Furthermore, exposure of developing embryos to sediments collected from sites in Louisiana caused similar molecular responses and induced developmental abnormalities that were consistent with well-known effects caused by PAHs. From these studies, we have clear evidence that these fish were exposed and responded to the toxic components of oil from the Deepwater Horizon Oil Spill (DHOS) via complex biochemical responses. These exposures persisted for more than a year following initial oiling, suggesting that fish from Louisiana marshes were exposed chronically to weathered oil [13]. Using these same oil-exposed fish [11], our primary objective was to determine whether trace metal signatures associated with the Macondo spill could be detected at elevated concentrations in the otoliths of *F*. *grandis*. This is essential proof-of-concept data to demonstrate the applicability of this approach across a diversity of inshore and offshore taxa of concern in the northern GOM. Additionally, we evaluated the chemical composition of the same otoliths to more broadly determine if other environmental perturbations in the GOM may have affected otolith elemental composition.

## Materials and Methods

### Ethics Statement

The fish used in this study were euthanized by severing the spinal cord. The protocol was approved by the Institutional Animal Care and Use Committee (IACUC) at Louisiana State University (Protocol Number: 10–098). Saltwater Scientific Collecting permits were provided by the LA Department of Wildlife.

### Fish Collections

**Fig 1:**
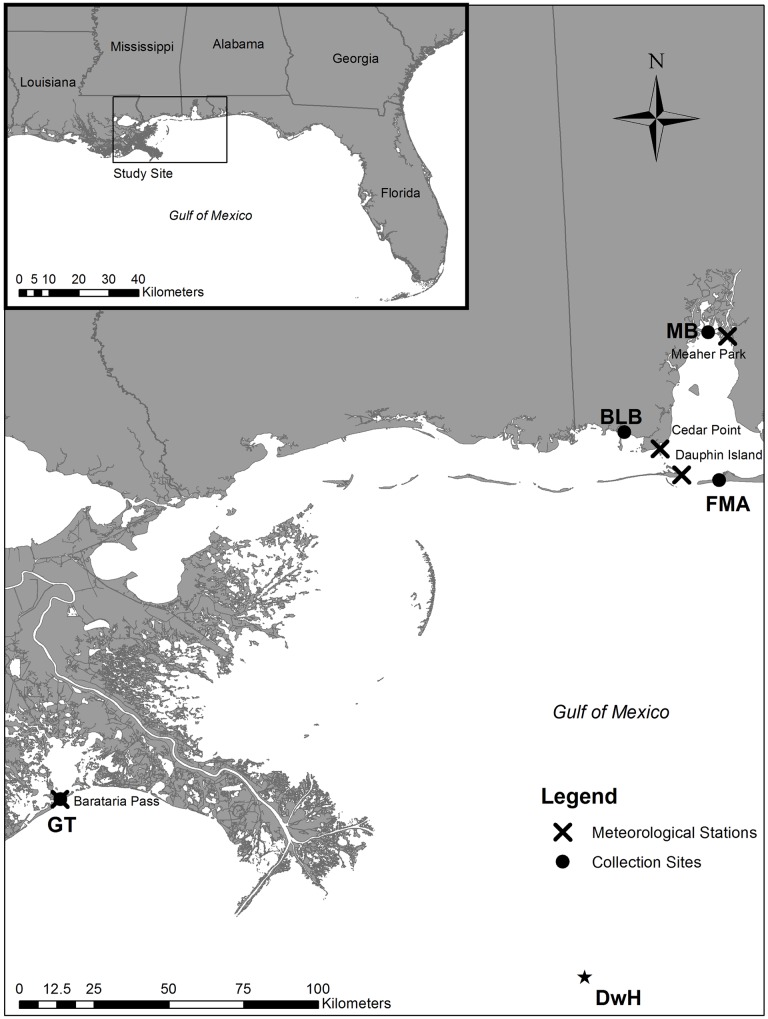
Map of study sites in the northern Gulf of Mexico. General (inset) and specific location of Gulf killifish (*Fundulus grandis*) collection sites in Grande Terre [GT], LA; Bayou La Batre, AL [BLB]; Mobile Bay, AL [MB]; and Fort Morgan, AL [FMA], throughout 2010 (May-August). The point labeled DwH indicates the site of the Deepwater Horizon explosion on April 20, 2010. The closest meteorological stations to the fish collection sites are marked by Xs.

We employed a BACI design to evaluate any changes in the chemical composition of otoliths associated with the Macondo spill. Male and female *Fundulus grandis* were collected, as reported in Whitehead et al. [11], using wire mesh traps placed along marsh edges in Grande Terre, Louisiana (GT); Bayou La Batre, Alabama (BLB); Mobile Bay, Alabama (MB); and Fort Morgan, Alabama (FMA) (Table 1, Fig 1). Whitehead et al. [11] dissected tissue samples from male fish *in situ* for morphological analysis and immunohistochemical analysis of CYP1A protein expression in gills, genome expression in the liver, and analytical chemistry (total PAHs). The remaining carcasses (57–99 mm total length, Table 1) were stored at -20°C until use for otolith microchemistry analysis (Table 1). Analytical chemistry of tissue samples was not sensitive for detecting oil pollution, yet biological responses were [11]; only fish from GT were directly exposed to Macondo oil and showed divergent genomic and physiologic responses coincident with, and diagnostic of, oil exposure [11,13].

**Table 1:** Gulf killifish (*Fundulus grandis*) collection sites, coordinates, meteorological stations (for temperature and salinity records), and sampling dates during 2010.

Collection Site	Location (Latitude, Longitude)	Meteorological Station (<1–10 km)	Killifish Collection Dates (No. of otoliths for chemical analysis)			Total Length (mm)	
			Pre-oil	Peak oil	Post-oil	Mean ± SEM	Size Range
Grand Terre Island, LA (GT)	29°16'22.93"N, 89°56'41.87"W	Barataria Pass at Grand Isle, LA	5/9/2010 (n = 6)	6/28/2010 (n = 6)	8/30/2010 (n = 6)	78.8 ± 2.4	61–97
Bayou La Batre, AL (BLB)	30°22'42.43"N, 88°14'47.42"W	Cedar Point, AL	5/2/2010(n = 6)	6/29/2010(n = 6)	9/1/2010(n = 6)	74.6 ± 1.7	57–90
Mobile Bay, AL (MB)	30°40'43.77"N, 87°59'40.06"W	Meaher Park, Mobile Bay, AL	5/5/2010 (n = 6)	6/30/2010 (n = 6)	no samples^a^ (n = 0)	84.1 ± 2.2	72–95
Fort Morgan, AL (FMA)	30°14'1.25"N, 87°57'40.84"W	Dauphin Island, AL	5/5/2010 (n = 6)	6/29/2010 (n = 6)	9/1/2010 (n = 6)	84.1 ± 2.2	70–99

^a^There are no post-oil samples from MB as the last fish collected at this site are from June (peak-oil).

FMA and GT are barrier islands and experience similar salinity regimes (>20). MB, on the other hand, experiences low salinities year-round (<10). BLB falls in the middle of this salinity continuum (~10–20). Fish from our four study sites were collected on May 1–9, 2010 (n = 6) before oil reached the coastline (pre-oil samples), on June 28–30, 2010 (n = 6) immediately following the peak of oil landfall in mid-June (peak-oil samples), and two months later on August 30-September 1, 2010 (post-oil samples, n = 6) (Table 1). No fish were collected in MB during the post-oil sampling event.

### Environmental Data

Trace elemental uptake is often influenced by both temperature and salinity. Therefore, the environmental conditions experienced by the fish in this study were evaluated. Mean values for monthly temperature and salinity in April-August, 2010 (i.e., the period most relevant for our collections), were obtained from USGS and NOAA meteorological stations in the vicinity of each sampling site (see Table 1). Reporting monthly means for both salinity and temperature is appropriate for otolith microchemistry studies because otoliths integrate environmental information over longer periods of time. For gene expression and many aspects of physiology with short-term responses [41], records of daily salinity at the time the fish were collected were appropriate and were reported in Whitehead et al. [11]. To characterize oil contamination (total PAHs), surface water samples were collected during the three sampling efforts at all sites after the grounding of oil, and sediment samples were collected within a month of the last sampling effort [11]. Analytical chemistry of surface water samples was not sensitive for detecting oil pollution during pre-, peak, and post-oil sampling efforts, but high PAH levels in sediments were detected at GT following oiling [11]. Of the field sites, only GT was directly oiled, which occurred after the first sampling time point (May 09, 2010) and prior to the second sampling time point (June 28, 2010). This was confirmed by satellite imagery, visual observation, biological responses, and sediment chemistry as reported in Whitehead et al. [11].

### Otolith Preparation

Frozen fish samples were transported to the Rutgers University Marine Field Station (IACUC Protocol No. 88–042), Tuckerton, NJ, where otoliths were dissected and prepared for microchemical analysis. All supplies used to handle the otoliths were washed in 10% nitric acid (Optima). Both pairs of sagittal otoliths were extracted using teflon tip forceps. Visible tissue was cleaned by gently rubbing the otoliths against a wet Kim Wipe (wet with MilliQ water, Ultrapure) to remove attached organics. Right sagittal otoliths were soaked in MilliQ water for 5 minutes. Water was removed and a 200-μl solution of buffered (0.05 N NaOH) hydrogen peroxide (15% H_2_O_2_) was added to each vial. Two minutes later, 200-μl of 1% nitric acid were added to each vial. After 5 minutes, the solution in the vials was removed. The otoliths were immediately rinsed three times with 200 μl of MilliQ water and then dried under a class-100 laminar flow hood (Air Control Inc.).

Otoliths were cut and polished to obtain a dorsoventral transverse section (~300 μm thick) that exposed the core. First, clean otoliths were embedded in Epothin 2 Epoxy Resin and Hardener. The anterior section of each otolith was cut along the transverse plane with a diamond saw (Hillquist Inc.) at approximately 200–300 μm from the core. This cut side was slightly polished and glued onto a petrographic slide using Epothin 2 Expoxy Resin and Hardener. The posterior side of the otolith was then cut and polished with a diamond polisher (Hillquist Inc.) at a distance of 50–100 μm from the core. Polishing films (Precision Fiber Products, Inc.) of 30, 9, and 3 μm were used to carefully remove otolith material until the core was reached and the surface of the otoliths was further smoothed using a Mircocloth fabric (Buehler) with MicroPolish solution (Buehler). Finally, the otolith cross sections were cleaned using a soft tooth brush soaked first in 15% buffered hydrogen peroxide, then 1% nitric acid, and finally rinsed three times using Milli Q water. All samples were dried under the laminar flow hood before being stored.

### Otolith Analysis

Otoliths were analyzed using laser-ablation inductively-coupled-plasma mass spectrometry (LAICPMS) at the University of North Carolina at Chapel Hill’s Mass Spectrometry Facility in September 2013. Otolith material was ablated using a Photon Machines Analyte G2 laser ablation unit (193 nm wavelength). Each otolith was sampled by ablating one 150-μm line along the most recent growth increments at the ventral edge of the otolith (4.2 mJ/cm^2^ intensity, 10-μm/s scan speed, and 110-μm spot size). Based on otolith growth data for *F*. *grandis* of similar sizes (Anthony Vastano, personal communication), the ablated portion of otoliths corresponded to approximately the last month before capture. Ablated material was transported from the laser unit using a mixture of helium (He) and argon (Ar) gases to a Thermo Scientific Element XR Inductively-Coupled-Plasma Mass Spectrometer (ICPMS). The isotopes of eight elements were recorded: calcium (^48^Ca), vanadium (^51^V), manganese (^55^Mn), nickel (^60^Ni), copper (^63^Cu), strontium (^88^Sr), barium (^138^Ba), and lead (^208^Pb). To prevent nickel interference, the ICPMS was fitted with an aluminum skimmer and sample cones (RA Chilton ICPMS Cones Ltd.). Elements were selected for analysis based on their potential use as indicators of oil (e.g., V and Ni; [42]) or pollution (e.g., Cu, Pb from terrestrial sources; [43]) exposure. The list also included elements traditionally used as environmental indicators of temperature and salinity, such as Sr and Ba [44,45], and sediment redox (Mn; [46]). Calcium was used as a universal internal standard to account for the amount of otolith material ablated. A glass standard spiked with trace elements (National Institute Standards and Technology [NIST]-612 glass reference material, [47]) was analyzed at the beginning and end of each day, as well as every time the laser sample chamber was opened to exchange samples (~ every 10–15 otoliths). To correct for daily machine drift, otolith element:Ca ratios were multiplied by a correction factor generated from the NIST 612 standard runs. The NIST 612 data were also used to measure analytical precision (% relative standard deviation, RSD) for each element: V (10.1%), Mn (11.1%), Ni (10.2%), Cu (10.3%), Sr (13.9%), Ba (14.1%), Pb (12.7%).

### Data Analysis

Data processing to determine elemental concentrations was conducted as described by Fodrie and Levin [48]. For each run, the mass spectrometer produced a chromatogram (counts over time). Detection limits for each element were defined as three standard deviations (SD) above the mean of the background. Detection limits (reported as element:Ca concentrations) were calculated by averaging data from 16 otoliths and were based on acquiring 100 million ^48^Ca counts per second: 0.04 μmol V /mol Ca; 0.16 μmol Mn/mol Ca); 0.50 μmol Ni/mol Ca); 0.36 μmol Cu/mol Ca); 0.25 μmol Sr/mol Ca); 0.02 μmol Ba/mol Ca); and 0.05 μmol Pb/mol Ca). For signals above detection limits, background signals were subtracted from sample signals, and the area under the chromatogram peak was calculated (total counts). Elemental signals below the detection limits threshold were assigned a random value between zero and the detection limit.

Following our BACI design, the effect of collection location (control reference vs. impact [i.e., oiled]) and time (before vs. after the grounding of oil at GT) on the elemental concentration of killifish otoliths was evaluated using two-way analysis of variance (ANOVA). Traditional BACI designs study one impact and one control location and do not replicate sites. Underwood [49] stressed the importance of sampling multiple control sites and multiple impacts sites when logistically possible, to increase the likelihood that observed differences are due to the impact in question. In this instance, field sites were established prior to the arrival of contaminating oil and we were not able to control the number of impacted or control sites as the trajectory of oil was highly patchy along northern GOM coasts. Oil made landfall at the GT site only, such that the remaining sites (BLB, MB and FMA) were considered unoiled reference sites. Thus, we considered our two-way analyses in three distinct ways: (1) averaging all data from BLB, MB and FMA in to an overall reference signature; (2) using only the data from FMA as the reference signature, given that FMA was most environmentally similar (e.g., salinity, temperature) to GT; and (3) using only data from BLB and FMA as the reference signature, in essence removing MB from our analyses since that site was most environmentally distinct from all other sites (low salinity), and post-spill data from MB were not available. Notably, the qualitative patterns we observed with respect to statistical and biological significance in otolith signals were conserved regardless of what approach we utilized for defining reference sites/specimens (i.e., regardless of statistical approach, patterns were similar; see S1, S2, S3 and S4 Tables). For simplicity, we hereafter present only the merged results of BLB and FMA as the reference signature.

Our final ANOVA model included site and sampling period as main factors, as well as the effect of the site x sampling period interaction term. This design is based on the hypothesis that temporal changes in otolith signatures at the oiled site (GT) before and after the grounding of oil should be distinct from temporal changes that occur at unoiled control sites [50]. Thus, the ANOVA term of most interest was the site x sampling period interaction.

Separate two-way ANOVAs were run for the seven element:Ca ratios that were evaluated. Furthermore, separate two-way ANOVAs were run to first compare the pre-oil vs. peak-oil periods, and then the pre-oil vs. post-oil periods (i.e., 14 total analyses). Because each of the element:Ca metrics across the two different temporal comparisons was considered a distinct, independent test of oil contact, we did not make corrections to experiment-wise alpha [51]. Rather, for each element and across all sampling periods, we considered *p* values (without denoting an arbitrary alpha), effect sizes, and variances to evaluate the strength of evidence for otolith-based markers of oil contact [52,53]. For the ANOVAs, all element:Ca data were Log (x + 1) transformed to meet the assumptions of normality and homoscedasticity, resulting in only a few modest violations for parametric tests (against which ANOVAs are largely robust). Additionally, the associations between two environmental parameters, salinity and temperature, and otolith Ba:Ca and Sr:Ca were also evaluated using linear regression (i.e., four separate analyses).

## Results

### Environmental Variables

**Fig 2:**
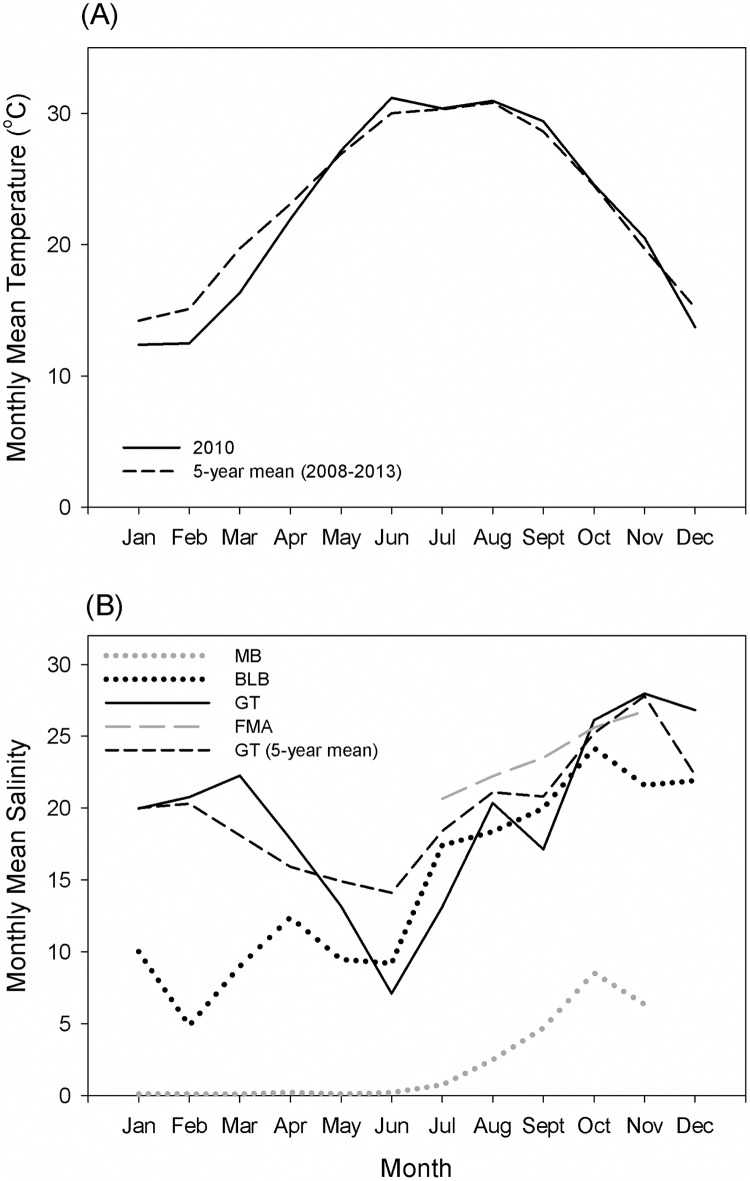
Monthly mean temperature and salinity at fish collection sites. (A) Temperature values were obtained from Barataria Bay Pass, LA (USGS 073802516) and were considered representatives of surface waters in the northern Gulf of Mexico. The solid line represents the values for GT in 2010 and the dashed line the values for the 5-year mean. (B) Salinity values were obtained from stations near collection sites, including Maeher Park, AL (MB), Cedar Point, AL (BLB), Barataria Pass, LA (GT), and Dauphin Island, AL (FMA). The solid line represents values for GT in 2010 and the dashed line the values for the 5-year mean obtained from Barataria Pass.

Surface water temperatures at GT, BLB, MB, and FMA in 2010 were consistent with the 5-year (2008–2013) seasonal mean for the northern Gulf of Mexico (Fig 2A). At all sites, mean temperatures were approximately 20°C during April, and experienced a seasonal increase to approximately 30°C during May 2010, and remained at an average of 30°C from June to August.

Salinity regimes for these four sites were variable (Fig 2B). Salinity levels near MB were the lowest. Monthly mean salinity in MB remained below 1 from April-July and rose slightly to 2.5 in August. The salinity at BLB fluctuated considerably between April and August (with daily values ranging from 2 to24). Monthly salinity averages were lowest from April to June (mean = 10) and highest in July-August (mean = 18). Near GT, monthly average salinity was 18 in April, decreased in May to 13, and reached the lowest average in June (7), before increasing to 13 in July. Notably, freshwater diversions in Louisiana which were increased in volume to slow the progression of oil into coastal habitats were evident at GT as revealed by the drop in salinity in June and August, which averaged 6 lower than the calculated 5 year mean (2008–2013) for that time of the year. Salinity data for FMA were not available for the first six months of 2010. However, FMA had the highest salinity levels of all four sites during July and August, fluctuating between 13 and 30 and averaging 22.

### Otolith Responses

**Fig 3:**
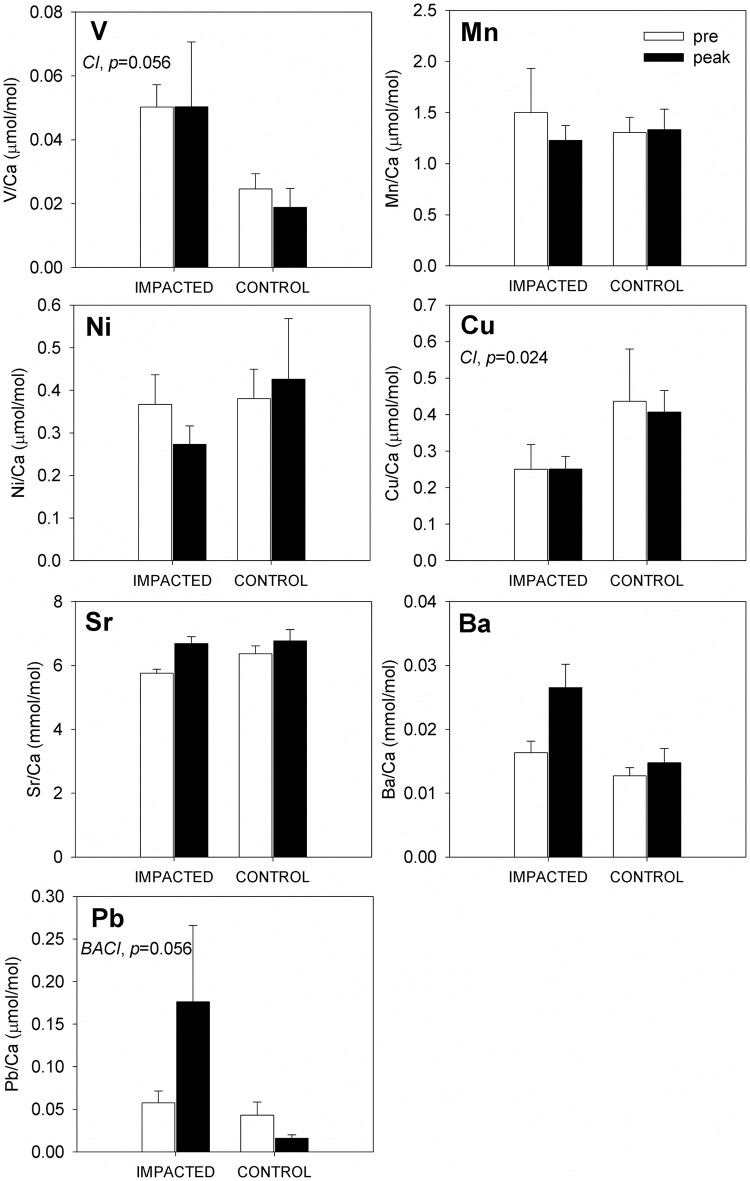
Composite of trace element concentrations in Gulf killifish (*Fundulus grandis*) otoliths. Fish were collected pre- (white bars) and peak (black bars) oil contamination at impacted (GT) and control sites (FMA, BLB). Each plot depicts the mean elemental concentrations (mean ± SEM) in μmol/mol (V, Mn, Cu, Pb) or mmol/mol (Sr and Ba) of several otolith replicates. Sample sizes for the impacted sites are n = 6 (pre) and n = 6 (peak) and for control sites n = 12 (pre) and n = 12 (peak). Before-after (pre vs. peak), impacted-control, and/or interaction effects are noted with a BA, CI, and or BACI respectively along with *p* values. ANOVA tables for the two-way, before-after control-impact (BACI) design are included in Table 2.

In the immediate aftermath of oil grounding at GT, changes in the otolith concentrations of Ba (moderate evidence) and Pb (strong evidence) were distinct from the concentrations in otoliths from fish at control sites (Table 2, Fig 3). Mean otolith Ba:Ca (mmol/mol) levels at GT increased by 40% between the pre-oil and peak-oil sampling, while otolith Ba:Ca concentrations among the control sites shifted negligibly over the same time. Statistical support for a meaningful, consistent interaction between site and time in otolith Ba:Ca signatures was not strong however (*p* = 0.112; Table 2). Similarly, mean otolith Pb:Ca signatures were similar between fish from GT and control sites during pre-oil sampling, and then diverged notably during peak-oil sampling. At GT, mean otolith Pb:Ca concentrations rose by over 300% through time (note large standard deviation), while otolith Pb:Ca concentrations of fish at control sites (collected at the same time as peak oiling at GT) fell to near 50% of pre-oil levels (Fig 3). Statistical evidence supported a marginally significant interaction between site and time on otolith Pb:Ca in our BACI design (*p* = 0.059; Table 2). Among all other elements, the only notable differences across space or time were as follows: (1) otolith V:Ca concentrations were consistently higher (approximately double) at GT relative to control sites, regardless of sampling period (main effect of site *p* = 0.056; Table 2); and (2) otolith Cu:Ca concentrations were consistently lower (approximately a 40% decline) at GT relative to control sites, regardless of sampling period (main effect of site *p* = 0.024; Table 2).

**Table 2:** ANOVA table for two factor BACI design, pre- vs. peak oil comparison.

Element	Source	Sum of Squares (SS)	Df	F	*p*
V	Time: BA	1.60x10^-5^	1	1.87	0.402
	Location: CI	0.001	1	129.53	0.056
	Interaction: BACI	8.557x10^-6^	1	0.65	0.800
	Error	0.004	32		
	Total		35		
Mn	Time: BA	0.001	1	2.298	0.371
	Location: CI	1.360E-5	1	0.053	0.855
	Interaction: BACI	0.000254	1	0.014	0.907
	Error	0.584	32		
	Total		35		
Ni	Time: BA	0.001	1	0.740	0.548
	Location: CI	0.002	1	1.206	0.470
	Interaction: BACI	0.002	1	0.249	0.621
	Error	0.247	32		
	Total		35		
Cu	Time: BA	0.000174	1	7.171	0.228
	Location: CI	0.017	1	691.615	0.024
	Interaction: BACI	2.434E-5	1	0.003	0.956
	Error	0.251	32		
	Total		35		
Sr	Time: BA	0.012	1	5.169	0.264
	Location: CI	0.003	1	1.136	0.480
	Interaction: BACI	0.002	1	0.830	0.369
	Error	0.088	32		
	Total		35		
Ba	Time: BA	5.391E-5	1	2.275	0.373
	Location: CI	8.650E-5	1	3.650	0.307
	Interaction: BACI	2.370E-5	1	2.676	0.112
	Error	0.000283	32		
	Total		35		
Pb	Time: BA	0.002	1	0.324	0.671
	Location: CI	0.008	1	1.578	0.428
	Interaction: BACI	0.005	1	3.840	0.059
	Error	0.042	32		
	Total		35		

BLB and FMA were used as the reference (control) signature. BA = before-after, CI = control-impact.

**Fig 4:**
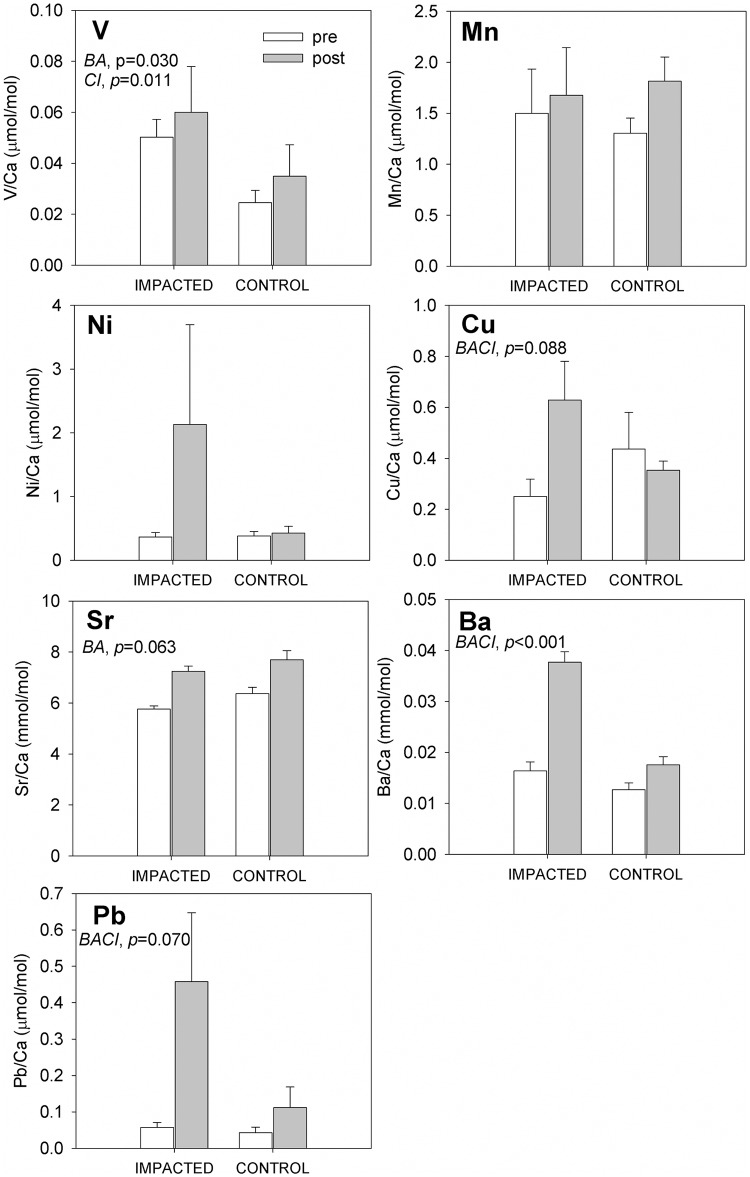
Composite of trace element concentrations in Gulf killifish (*Fundulus grandis*) otoliths. Fish were collected pre- (white bars) and post (gray bars) oil contamination at impacted (GT) and control sites (FMA, BLB). Each plot depicts the mean elemental concentrations (mean±SEM) in μmol/mol (V, Mn, Cu, Pb) or mmol/mol (Sr and Ba) of several otolith replicates. Sample sizes for the impacted sites are n = 6 (pre) and n = 6 (post) and for control sites n = 12 (pre) and n = 12 (post). Before-after (pre vs. post), impacted-control, and/or interaction effects are noted with a BA, CI, and or BACI respectively along with p values. ANOVA tables for the two-way before-after control-impact (BACI) design are included in Table 3.

Two months later, at the “post-oil" sampling, otolith concentrations of Ba (strong evidence) and Pb (strong evidence) were distinct between fish from oiled and reference sites (Table 3, Fig 4). Additionally, otolith concentrations of Cu (strong evidence) and Ni (very modest evidence) were suggestive of changes at GT that were different from patterns recorded across control sites. Mean otolith Ba:Ca concentrations more than doubled between pre-oil and post-oil sampling at the GT site, while otolith Ba:Ca concentrations among the control sites increased by only slightly between pre-oil and post-oil sampling. Moreover, the absolute change in otolith Ba:Ca at GT (a 0.02 mmol/mol increase) was five-times greater than the change observed at control sites (a 0.004 mmol/mol increase). Statistical support for an interaction between site and time in otolith Ba:Ca signatures was compelling (*p*<0.001; Table 3). As before, Pb:Ca signatures were similar between GT and control fish during pre-oil sampling, and then diverged notably during post-oil sampling. At GT, mean otolith Pb:Ca concentrations rose by over 800% through time (note large standard deviation), while Pb:Ca concentrations at control sites only doubled over the same period. Again, statistical evidence supported a marginally significant interaction between site and time on Pb:Ca in our BACI test (*p* = 0.070; Table 3). While mean otolith Cu:Ca concentrations were nearly twice as high at control sites relative to GT before the grounding of oil, this pattern was reversed with Cu:Ca concentrations being twice as high at the oiled site relative to control sites during the post-oil sampling (statistical test for a site x time interaction, *p* = 0.088; Table 3). This result was driven primarily by changes over time at GT, as otolith Cu:Ca levels at control sites were relatively stable between pre-oil and post-oil sampling. Mean otolith Ni:Ca concentrations were also elevated at GT post-spill (i.e., a 400% increase relative to pre-oil levels) in a manner not reflected at control sites (where Ni:Ca levels remained stable through time). However, statistical evidence for a site x time interaction in Ni:Ca was weak (*p* = 0.185; Table 3), and we note that the elevated post-oil mean at GT was driven by an outlier. Again, V:Ca concentrations were consistently higher at GT than all other sites (approximately double; main effect of site *p* = 0.011), and over time we recorded a 25% increase in V:Ca evenly across sites (main effect of time *p* = 0.030, Table 3). Sr:Ca levels also increased by roughly 25% between pre-oil and post-oil sampling regardless of site type (main effect of time *p* = 0.063; Table 3).

**Table 3:** ANOVA table for two-factor BACI design, pre- vs. post-oil comparison.

Element	Source	Sum of Squares (SS)	Df	F	*p*
V	Time: BA	1.2E-4	1	449.69	0.030
	Location: CI	0.001	1	3342.39	0.011
	Interaction: BACI	2.672E-7	1	0.001	0.971
	Error	0.006	32		
	Total		35		
Mn	Time: BA	0.025	1	5.262	0.262
	Location: CI	0.003	1	0.676	0.562
	Interaction: BACI	0.005	1	0.208	0.651
	Error	0.733	32		
	Total		35		
Ni	Time: BA	0.064	1	1.233	0.467
	Location: CI	0.050	1	0.963	0.506
	Interaction: BACI	0.052	1	1.836	0.185
	Error	0.906	32		
	Total		35		
Cu	Time: BA	0.020	1	0.747	0.546
	Location: CI	0.001	1	0.055	0.854
	Interaction: BACI	0.027	1	3.094	0.088
	Error	0.274	32		
	Total		35		
Sr	Time: BA	0.049	1	100.558	0.063
	Location: CI	0.006	1	12.078	0.178
	Interaction: BACI	4.84E-4	1	0.172	0.681
	Error	0.090	32		
	Total		35		
Ba	Time: BA	2.46E-4	1	2.556	0.356
	Location: CI	2.03E-4	1	2.107	0.384
	Interaction: BACI	9.634E-5	1	19.219	<0.001
	Error	1.60E-4	32		
	Total		35		
Pb	Time: BA	0.040	1	2.202	0.378
	Location: CI	0.023	1	1.287	0.460
	Interaction: BACI	0.018	1	3.526	0.070
	Error	0.165	32		
	Total		35		

Data from GT were used as the impact signature and data from BLB and FMA were used as the reference (control) signature. BA = before-after, CI = control-impact.

**Fig 5:**
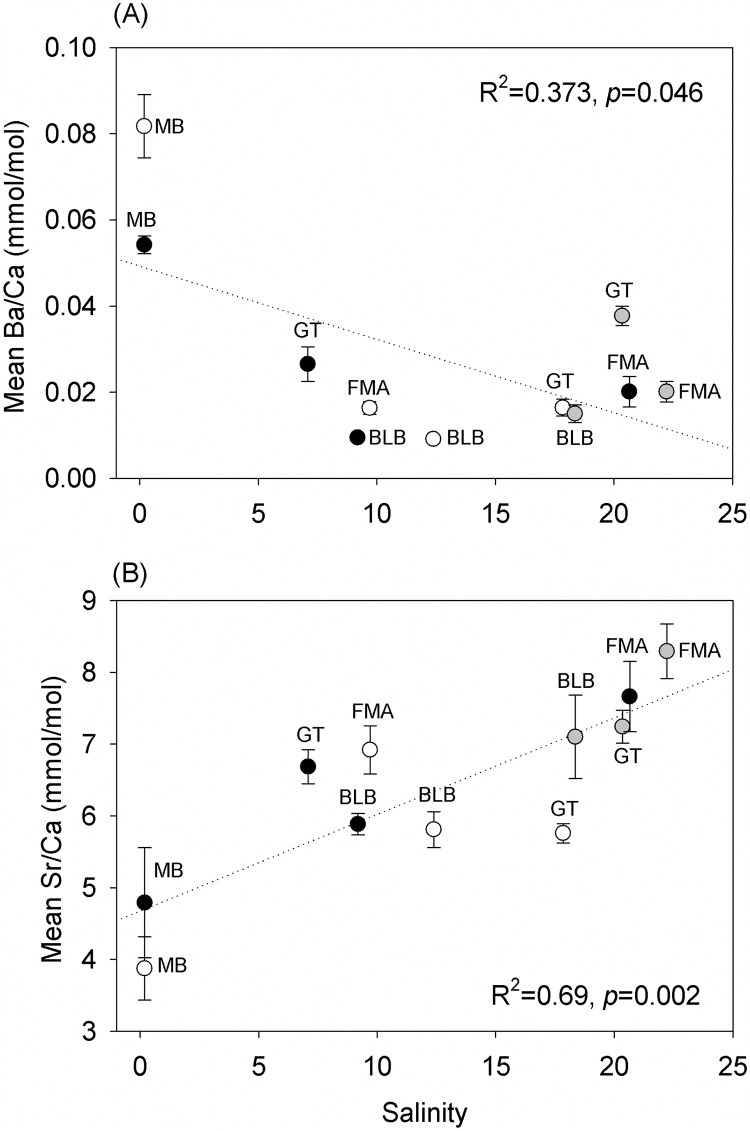
Relationships between (A) Ba and (B) Sr concentrations in in Gulf killifish (*Fundulus grandis*) otoliths. Fish were collected in MB, FMA, GT, and BLB. Salinity averages include the months preceding pre-oil (white circles), peak (black circles), and post-oil (gray circles) collections. Because salinity data could not be obtained for May-Jun at FMA, salinity measured at the time of collection was used for this plot.

Changes in Ba and Sr concentrations were associated with temporal and spatial changes in salinity. The relationship between salinity and Ba concentration was negative (R^2^ = 0.373, *p* = 0.046) (Fig 5A), while the relationship to Sr concentration was positive (R^2^ = 0.69, *p* = 0.002) (Fig 5B). No significant relationship between Ba concentrations and temperature (R^2^ = 0.02, *p* = 0.677) or Sr concentrations and temperature (R^2^ = 0.105, *p* = 0.330) were detected.

## Discussion

We detected differences in the chemical composition of *F*. *grandis* otoliths collected before and after the Macondo oil spill at impacted vs. control sites, but not necessarily in the elements that we initially expected. Spatial and temporal patterns in otolith elemental concentration varied according to the element being examined, the sites, and the time of fish collection (pre, peak, or post-oil). There were no temporal changes (before-after [BA] effects) when we compared otoliths collected before the oil reached the marshes in May (pre-oil) and a few days after it reached the marshes in late-June (peak-oil). However, spatial differences (control-impact [CI] effects) in both V and Cu concentrations were detected, but there was no interaction effect between site and time (no BACI effect). The only exception was Pb, where an increase in concentration was observed only at the impacted site (GT) following oiling, but not at any reference sites (interaction effect). Most importantly, two months following oiling (post-oil), there was an observed increase in the concentrations of Cu, Ba, and Pb only in otoliths at the impacted site (interaction effect), which could indicate a signature of DHOS oil or another distinction for fish from GT in environmental exposure to these elements.

### V and Ni: Putative Oil Markers

Crude oil is composed primarily of C, H, O, N, S, V, Ni, and Fe [42]. The initial goal of this study was to determine whether exposure to crude oil, which has typically higher concentrations of V and Ni than seawater, would result in an increase in concentration of these two elements (previously highlighted as oil markers) in otoliths of fish known to have been exposed to oil. Based on our analysis of these data, there was no clear, consistent evidence of an increase in V or Ni in the otoliths of oil-impacted killifish at the GT site or reference sites. This is perhaps not surprising, since V and Ni are not always consistently increased in tissues following exposure to oil [54]. We do acknowledge that in comparing pre-oil vs. post-oil samples, mean Ni concentrations increased at GT (driven by an outlier) while no increase was observed at our control sites. However, Ni loads in otoliths of individuals collected at GT post-oiling were highly variable and, thus, it is unclear if Ni can serve as a reliable marker of oil exposure. Notably, V concentrations in GT otoliths were already higher than in BLB and FMA before the oil spill, which may reflect a long history (~70 years) of oil exploration and previous spills in Louisiana (US Minerals Management Service and US Coast Guard data in Turner et al. [55]) relative to Alabama.

We consider several explanations for the mismatch between documented genomic and physiological responses of killifish and the absence of elevated V and Ni in the otoliths of oil-impacted fish. First, the most likely explanation is that the trace elements that traditionally serve as oil indicators (i.e. V and Ni) occur in low concentrations in Macondo oil relative to other crude oils. V and Ni are the most abundant metals in petroleum, but the concentrations vary among oil reservoirs around the world [29]. Depending on the source, concentrations of V can reach up to 1580 ppm and Ni up to 340 ppm [56]. Relative to other crude oils, the concentrations of V and Ni in Macondo oil are relatively low, <1.0–1.7 ppm, and <2.0–7.3 ppm, respectively [33,34].

A recent study by Nelson et al. [36] acknowledged the chemically “light” nature of Macondo oil in terms of trace metal loads. In that study, the researchers found distinct regional signatures in the chemical composition of *F*. *grandis* otoliths collected two years post-spill across the northern GOM, but no statistically significant differences in the otoliths of fish from paired oiled versus unoiled sites. However, nearly all of their individuals were spawned after the Macondo oil reached shore, based on maximum age estimates of 23 months [57]. Thus, it was unclear if the animals used in those experiments had in fact been exposed to oil. Our results agree with Nelson et al. [36] and strengthen the conclusion that Ni and V markers do not document a history of exposure to Macondo oil in *F*. *grandis* otoliths.

Second, following exposure to Macondo oil, fish growth rates and physiological performance could have been impacted, decreasing the rate of incorporation of Ca and other divalent cations into the otoliths. Direct exposure to oil and other toxicants does impact the metabolic and energetic function of fish [58]. Exposure to oil can alter cell membrane structure and function and can lead to abnormal gill morphology and function [59], potentially affecting respiration and metabolic potential. Moreover, the metabolic cost associated with PAH metabolism is likely high, potentially requiring reallocation of energy from growth, reproduction, etc. [58]. Reduced weight and prey-capturing ability has been associated with mercury contamination in Atlantic killifish (*F*. *heteroclitus*), indicating that a metabolic cost associated with toxicity is manifested in growth [60], and the expression of several genes associated with metabolic activity were divergent in these fish [11,13]. Further, decrease in otolith growth due to crude oil exposure has been observed in juvenile sea bass [61]. It follows that because the fish from GT in the present study clearly exhibited physiological responses to oil pollution, it is possible that somatic or otolith growth could have been reduced, and as a result, oil signatures were not incorporated into the otoliths.

Alternatively, an increase in otolith growth due to crude oil has also been reported following previous oil spills. A decrease in abundance of large Atlantic killifish (*F*. *heteroclitus*) following an oil spill in the Arthur Kill, New Jersey, was associated with an increase in food resources available to young-of-the-year (small) Atlantic killifish one year after the spill, likely explaining an increase in fish abundance and faster growth rates among smaller size classes [62]. However, fish assemblage comparisons conducted two years after the Macondo oil spill in Barataria Bay, Louisiana, indicate that there was no significant difference in the length frequency distributions of *F*. *grandis* in oiled vs. unoiled marshes [17]. Therefore, there is no evidence that *F*. *grandis* growth rates were affected in a manner that explains our otolith marker results.

Third, increases in levels of Ni and V in the otolith may be delayed relative to other elements based on the pathway of incorporation. For instance, V may only be bioavailable (in aqueous phase) following microbial degradation of oil [63]. Incorporation of these elements into higher trophic organisms may therefore be different because of physiological or perhaps food-web filters. For example, bivalve tissues and shells monitored for increased levels of V and Ni following the “*Erika*” tanker oil spill in France exhibited an increase in V only after five months, while elevated Ni levels persisted a year after oiling [31]. Although elemental profiles are generally an indication of surrounding water composition, food sources play a role in the accumulation of some of the elements that become incorporated into the otolith [64]. Indeed, temporal differences in the accumulation of V and Ni in otoliths can be attributed to the food habits of different taxa (e.g., marsh periwinkles, mussels, dogwelks; [30]. As adults, *F*. *grandis* feed on a variety of resources, including fiddler crabs, amphipods, tanaids, marsh periwinkles, and polycheates [65]. Evidence suggests that while some of these resources were suppressed by DHOS oil exposure (e.g. fiddler crabs), others were not (e.g., marsh periwinkles) [66], and oil can remain bound in sediments and associated food sources for years [13,67,68]. Killifish from this study showed high CYP1A expression in the intestine of GT fish for over one year following landfall of oil, indicating that these fish were likely exposed to PAHs through the diet or from inadvertent sediment consumption during feeding [13]. Still, it is unclear to what extent food sources could contribute to metal accumulation in otoliths over time in fish exposed to oil from the DHOS. To date, we have found no evidence in the literature to suggest that trace metals associated with the Macondo oil spill are present in higher concentrations in marsh food webs. For instance, trace element concentrations in oyster shells exposed to oil also did not corroborate oil exposure [69].

### Cu and Pb: Freshwater Diversion or Oil Markers

Notable increases in the concentrations of Cu and Pb were detected in the otoliths of fish collected two months after oil landfall at the GT impacted site (BACI effect). GT was not only oiled in the summer of 2010, but also experienced an unprecedented freshwater diversion in an effort to flush oil from the Barataria watershed. This influx of fresh water was released from the Mississippi River at six times the normal discharge volume, lasted from late-April to late-August, and reduced salinity in Barataria Bay well below typical values [70]. Therefore, it is possible that elevated Cu and Pb concentrations in fish otoliths could be associated with either the freshwater diversions (river source) or oil exposure (offshore source), or a combination of both sources and other contamination.

Historically, the main sources of Cu and Pb in the Mississippi River have been municipal wastewaters and mining (in addition to natural sources). Accordingly, these elements are both monitored because they can be indicators of pollution (e.g., [71,72]). Measurable traces of dissolved Cu (2 ppb) and Pb (<0.1 ppb) have been documented in Mississippi River waters [73]. However, their presence in estuaries is longstanding, where significant Pb loads in southern Louisiana are attributed to oil refinery effluent (Pb concentrations of 20 to 14,245 ppm in wetlands; [74]). In addition, some elements may be more bioavailable in lower salinities. For example, the accumulation of Cu in tissues at lower salinities has been reported for the congener *F*. *heteroclitus* [75]. By contrast to Mississippi River waters, Cu and Pb concentrations in Macondo oil are 0.5 and 0.3 ppm, respectively, and oil mousse samples collected near salt marshes in Mississippi were only slightly higher in concentration (closer to 3.3 and 1.5 ppm, respectively) [33]. Yet, Hanson and Zdanowicz [76] argued that organic contaminant exposure (PAHs) may also affect Cu levels in otoliths, as it is known to affect hepatic Cu concentration [77]. Following the *Prestige* oil spill in Spain, Cu and Pb concentrations in seabird feathers were two and five times higher than pre-spill levels, respectively, but returned to previous background concentrations after three years [54]. In our study, Cu and Pb concentrations in otoliths from GT fish sampled post-oil were also two and five times higher, respectively, than pre-oil samples, indicating that increased Cu and Pb in the otoliths could be indicative of oil exposure in GT fish. Notably, V and Ni were not detected in seabird feathers in the Moreno et al. [54] study, thus corroborating our results that do not support the utility of V or Ni as indicators of DHOS oil exposure in otoliths of Gulf fish species.

### Sr and Ba: Oil Signature or Record of Freshwater Diversions

Strontium and barium are commonly employed as proxies for salinity and temperature exposure history. As notes above, freshwater diversions in southern Louisiana were intended to slow the entry of oil into coastal areas [78]. At the USGS station in Barataria Pass (<200 meters from the GT collection site), very low salinity was recorded: almost 10 units below the 5-year mean (Fig 2B). In the present study, an increase in Ba concentration in fish otoliths was negatively correlated with salinity. The uptake of Ba in the otolith matrix can be regulated by (1) salinity, (2) temperature, and/or the (3) availability of Ba in the water column [79]. Ba has been negatively related to salinity in juvenile Atlantic croaker (*Micropogonias undulatus*) [79] and striped bass [80]. Therefore, the increase in Ba concentrations in otoliths from fish collected in GT may have been due to the low salinity derived from the freshwater diversions. Sr uptake is also associated with salinity, but the relationship is expected to be positive (Sr decreases with a decrease in salinity, e.g., Fig 2b). Therefore, our Sr observations (increase following freshwater diversion) may suggest that the change in Ba was not exclusively associated with lower salinities. Alternatively, the diversions could have resulted in an increase in Ba in the water column, as well as an increase in other trace elements. Multiple trace elements are found in higher concentrations in the Mississippi River and the northern Gulf of Mexico [55]. Joung and Shiller [81] highlighted the importance of considering both natural and anthropogenic sources of Ba when studying the Ba-salinity relationship in this region.

It is important to note that salinity measured at the time the fish were collected did not correlate with gene expression in the Whitehead et al. [11] study and the patterns of gene expression were not a result of changes in salinity, but exposure to oil. Consideration of monthly means for both salinity and temperature is appropriate for otolith microchemistry because environmental patterns are integrated into the otolith over longer periods of time. By contrast, for gene expression and many aspects of physiology with short-term responses [41], monthly means are not relevant. Moreover, environmental parameters like salinity are not necessarily associated with stress indicators, but PAHs solubility is higher at lower salinities [82] and this can enhance PAH-induced mortality (reviewed by Whitehead et al. [83]).

Crude oil also contains trace concentrations of Sr and Ba [29]. However, Sr and Ba concentrations in Macondo oil are similar to seawater [34]. While barite (barium sulfate [BaSO_4_]) is present in the drilling muds used to stem the flow from the Macondo blowout, it is unlikely to explain the higher Ba signal in the otoliths of *F*. *grandis* collected at MB. Furthermore, it seems unlikely that drilling-mud-derived Ba could have reached coastal estuaries at concentrations high enough to explain chemical signatures observed at GT (or MB). Barium from discharge drilling muds is a concern for benthic communities near areas of high use, and can persist at high concentrations for decades following drilling [72]. However, barite has a low solubility in water [84]. Others reported high concentrations of Ba within the oil plume (1000–1300 m deep) near the Macondo well, but the concentration of Ba peaked 6 km away from the well [34]. Considering that GT is approximately 150 km from the blowout, it is unlikely that the drilling muds used at the Macondo well contributed to the high Ba content at the GT site.

### Limitations of the Study

A long history of oil contamination in Louisiana, the nature of the Macondo oil spill, and the unpredictable trajectory of the oil to coastal marshes limited some aspects of this study. First, marshes in Louisiana have been exposed to oil contamination due to small scale oil spills and refinery effluent for over 70 years [55]. Therefore, we speculate that background levels of some trace elements associated with oil (e.g., V in this study) may be higher in these marshes than in other areas of the Gulf of Mexico, complicating our ability to conduct large-scale, control-impact comparative research. Second, the interpretation of these results is also limited because only one of the sites sampled pre-oil was also (a) directly contaminated by oil and (b) exposed to freshwater diversions. Ideally, fish would have been collected at different sites across coastal Louisiana that included both areas directly contaminated by oil and not impacted by freshwater diversions as well as sites affected by freshwater diversions but not oil contamination. However, it was not possible to predict where these sites would be at the time the study was initiated.

## Conclusions

The functional resilience and integrity of the Gulf of Mexico is being perturbed by both local (e.g., shoreline development; [85] and regional (climate change; [86]) stressors. Coastal Louisiana, in particular, is susceptible to a variety of oil impacts due to a long history of oil production and refinery activity [87], but also compounding stressors such as hurricanes [88,89], sea-level rise [90,91], large-scale freshwater diversions [10,92], and coastal subsidence from reduced sedimentation associated with the channelization of the Mississippi River, fossil fuel extraction, and marsh compaction [93]. Temporal fluctuations in water chemistry are also common. Despite known oil exposure in GT *F*. *grandis*, and the utility of otolith microchemistry in assessing oil exposure from other spills (e.g., *Prestige Oil Spill*: [28]), the use of otolith microchemistry for detecting oil exposure is complicated by the nature of the DHOS oil (source and weathering), the large geographical region over which oil was spilled, the long period of oiling, and re-oiling events. Moreover, other environmental stressors such as salinity change are likely recorded in hard body parts of GOM species (e.g., fish otoliths, oyster shells). In the present study, we detected multi-elemental differences across space and time. Low salinity records indicate changes in Ba concentrations may have been, in part, due to freshwater diversions, designed to halt the ingress of oil. Changes in Cu and Pb concentrations may also be linked to land pollution via freshwater diversions and/or be indicative of oil exposure. Our results highlight the potentially complex and indirect effects of the Macondo oil spill and human responses oil spills in Gulf of Mexico ecosystems and emphasize the need to consider multiple stressors acting simultaneously on inshore fish communities.

## Supporting Information

S1 Table ANOVA table for two factor BACI design, pre- vs. peak oil comparison. Data from GT were used as the impact signature and data from BLB, MB, and FMA were used as the reference (control) signature. BA = before-after, CI = control-impact.
(DOCX)

S2 Table ANOVA table for two-factor BACI design, pre- vs. post-oil comparison. Data from GT were used as the impact signature and data from BLB, MB, and FMA were used as the reference (control) signature. BA = before-after, CI = control-impact.
(DOCX)

S3 Table ANOVA table for two factor BACI design, pre- vs. peak oil comparison. Data from GT were used as the impact signature and data from FMA were used as the reference (control) signature. BA = before-after, CI = control-impact.
(DOCX)

S4 Table ANOVA table for two-factor BACI design, pre- vs. post-oil comparison. Data from GT were used as the impact signature and data from FMA were used as the reference (control) signature. BA = before-after, CI = control-impact.
(DOCX)
