# Supplementary material for: Is Exposure to Macondo Oil Reflected in the Otolith Chemistry of Marsh-Resident Fish?
Source: PLoS One. 2016 Sep 28;11(9):e0162699. doi: 10.1371/journal.pone.0162699 (PMC5040417; doi:10.1371/journal.pone.0162699)
Supplement: S1 Table — Data from GT were used as the impact signature and data from BLB, MB, and FMA were used as the reference (control) signature. BA = before-after, CI = control-impact. (DOCX) [file pone.0162699.s001.docx]

**S1 Table. ANOVA table for two factor BACI design, pre- vs. peak oil comparison.**

| **Element** | **Source** | **Sum of Squares (SS)** | **df** | **F** | ***p*** |
| --- | --- | --- | --- | --- | --- |
| V | Time : BA | 7.914E-6 | 1 | 2.839 | 0.341 |
|  | Location: CI | 0.001 | 1 | 537.886 | 0.027 |
|  | Interaction: BAxCI | 2.788E-6 | 1 | 0.028 | 0.869 |
|  | Error | .004 | 44 |  |  |
|  | Total |  | 47 |  |  |
| Mn | Time : BA | 0.003 | 1 | 23.455 | 0.130 |
|  | Location: CI | 0.281 | 1 | 2289.32 | 0.013 |
|  | Interaction: BAxCI | 1.2E-4 | 1 | 0.002 | 0.968 |
|  | Error | 3.416 | 44 |  |  |
|  | Total |  | 47 |  |  |
| Ni | Time : BA | 0.017 | 1 | 9.099 | 0.204 |
|  | Location: CI | 0.007 | 1 | 3.596 | 0.309 |
|  | Interaction: BAxCI | 0.002 | 1 | 0.110 | 0.742 |
|  | Error | 0.738 | 44 |  |  |
|  | Total |  | 47 |  |  |
| Cu | Time : BA | 0.001 | 1 | 0.559 | 0.591 |
|  | Location: CI | 0.015 | 1 | 12.015 | 0.179 |
|  | Interaction: BAxCI | 0.001 | 1 | 0.155 | 0.696 |
|  | Error | 0.344 | 44 |  |  |
|  | Total |  | 47 |  |  |
| Sr | Time : BA | 0.005 | 1 | 0.566 | 0.589 |
|  | Location: CI | 0.012 | 1 | 1.304 | 0.458 |
|  | Interaction: BAxCI | 0.009 | 1 | 0.911 | 0.345 |
|  | Error | 0.431 | 44 |  |  |
|  | Total |  | 47 |  |  |
| Ba | Time : BA | 1.7E-4 | 1 | 127056.4 | 0.002 |
|  | Location: CI | 1.6E-4 | 1 | 122772.7 | 0.002 |
|  | Interaction: BAxCI | 1.313E-9 | 1 | 1.2E-5 | 0.997 |
|  | Error | 0.005 | 44 |  |  |
|  | Total |  | 47 |  |  |
| Pb | Time : BA | 0.002 | 1 | 0.539 | 0.597 |
|  | Location: CI | 0.010 | 1 | 2.134 | 0.382 |
|  | Interaction: BAxCI | 0.005 | 1 | 4.762 | 0.034 |
|  | Error | 0.043 | 44 |  |  |
|  | Total |  | 47 |  |  |

Data from GT were used as the impact signature and data from BLB, MB, and FMA were used as the reference (control) signature. BA = before-after, CI=control-impact.
