# Supplementary material for: Is Exposure to Macondo Oil Reflected in the Otolith Chemistry of Marsh-Resident Fish?
Source: PLoS One. 2016 Sep 28;11(9):e0162699. doi: 10.1371/journal.pone.0162699 (PMC5040417; doi:10.1371/journal.pone.0162699)
Supplement: S2 Table — Data from GT were used as the impact signature and data from BLB, MB, and FMA were used as the reference (control) signature. BA = before-after, CI = control-impact. (DOCX) [file pone.0162699.s002.docx]

**S2 Table. ANOVA table for two-factor BACI design, pre- vs. post-oil comparison**.

| **Element** | **Source** | **Sum of Squares (SS)** | **df** | **F** | ***p*** |
| --- | --- | --- | --- | --- | --- |
| V | Time : BA | 1.8E-4 | 1 | 22.571 | 0.132 |
|  | Location: CI | 0.001 | 1 | 133.32 | **0.055** |
|  | Interaction: BAxCI | 8.2E-6 | 1 | 0.047 | 0.829 |
|  | Error | 0.007 | 38 |  |  |
|  | Total |  | 41 |  |  |
| Mn | Time : BA | 0.011 | 1 | 0.286 | 0.687 |
|  | Location: CI | 0.107 | 1 | 2.739 | 0.346 |
|  | Interaction: BAxCI | 0.039 | 1 | 0.591 | 0.447 |
|  | Error | 2.511 | 38 |  |  |
|  | Total |  | 41 |  |  |
| Ni | Time : BA | 0.041 | 1 | 0.478 | 0.615 |
|  | Location: CI | 0.030 | 1 | 0.346 | 0.662 |
|  | Interaction: BAxCI | 0.086 | 1 | 2.361 | 0.133 |
|  | Error | 1.378 | 38 |  |  |
|  | Total |  | 41 |  |  |
| Cu | Time : BA | 0.018 | 1 | 0.559 | 0.591 |
|  | Location: CI | 0.001 | 1 | 0.024 | 0.901 |
|  | Interaction: BAxCI | 0.032 | 1 | 3.455 | 0.071 |
|  | Error | 0.352 | 38 |  |  |
|  | Total |  | 41 |  |  |
| Sr | Time : BA | 0.081 | 1 | 67.74 | 0.077 |
|  | Location: CI | 4.6E-4 | 1 | 0.388 | 0.645 |
|  | Interaction: BAxCI | 0.001 | 1 | 0.202 | 0.656 |
|  | Error | 0.225 | 38 |  |  |
|  | Total |  | 41 |  |  |
| Ba | Time : BA | 5.9E-5 | 1 | 0.172 | 0.750 |
|  | Location: CI | 3.8E-5 | 1 | 0.112 | 0.795 |
|  | Interaction: BAxCI | 3.4E-4 | 1 | 9.366 | 0.004 |
|  | Error | 0.001 | 38 |  |  |
|  | Total |  | 41 |  |  |
| Pb | Time : BA | 0.045 | 1 | 2.539 | 0.357 |
|  | Location: CI | 0.027 | 1 | 1.510 | 0.435 |
|  | Interaction: BAxCI | 0.018 | 1 | 4.048 | 0.051 |
|  | Error | 0.166 | 38 |  |  |
|  | Total |  | 41 |  |  |

Data from GT were used as the impact signature and data from BLB, MB, and FMA were used as the reference (control) signature. BA = before-after, CI=control-impact.
