# Supplementary material for: Is Exposure to Macondo Oil Reflected in the Otolith Chemistry of Marsh-Resident Fish?
Source: PLoS One. 2016 Sep 28;11(9):e0162699. doi: 10.1371/journal.pone.0162699 (PMC5040417; doi:10.1371/journal.pone.0162699)
Supplement: S3 Table — Data from GT were used as the impact signature and data from FMA were used as the reference (control) signature. BA = before-after, CI = control-impact. (DOCX) [file pone.0162699.s003.docx]

**S3 Table. ANOVA table for two factor BACI design, pre- vs. peak oil comparison.**

| **Element** | **Source** | **Sum of Squares (SS)** | **df** | **F** | ***p*** |
| --- | --- | --- | --- | --- | --- |
| V | Time : BA | 1.1E-5 | 1 | 1.965 | 0.394 |
|  | Location: CI | 0.001 | 1 | 95.32 | 0.065 |
|  | Interaction: BAxCI | 5.4E-6 | 1 | 0.029 | 0.866 |
|  | Error | 0.004 | 20 |  |  |
|  | Total |  | 23 |  |  |
| Mn | Time : BA | 0.009 | 1 | 2.429 | 0.363 |
|  | Location: CI | 0.003 | 1 | 0.852 | 0.525 |
|  | Interaction: BAxCI | 0.004 | 1 | 0.190 | 0.668 |
|  | Error | 0.408 | 20 |  |  |
|  | Total |  | 23 |  |  |
| Ni | Time : BA | 0.001 | 1 | 0.957 | 0.507 |
|  | Location: CI | 4.9E-6 | 1 | 0.004 | 0.961 |
|  | Interaction: BAxCI | 0.001 | 1 | 0.258 | 0.617 |
|  | Error | 0.099 | 20 |  |  |
|  | Total |  | 23 |  |  |
| Cu | Time : BA | 0.008 | 1 | 1.181 | 0.474 |
|  | Location: CI | 0.001 | 1 | 0.171 | 0.750 |
|  | Interaction: BAxCI | 0.007 | 1 | 2.00 | 0.173 |
|  | Error | 0.068 | 20 |  |  |
|  | Total |  | 23 |  |  |
| Sr | Time : BA | 0.013 | 1 | 26.85 | 0.121 |
|  | Location: CI | 0.020 | 1 | 42.75 | 0.097 |
|  | Interaction: BAxCI | 4.5E-4 | 1 | 0.255 | 0.619 |
|  | Error | 0.037 | 20 |  |  |
|  | Total |  | 23 |  |  |
| Ba | Time : BA | 5.3E-5 | 1 | 4.795 | 0.273 |
|  | Location: CI | 1.1E-5 | 1 | 1.039 | 0.494 |
|  | Interaction: BAxCI | 1.1E-5 | 1 | 1.190 | 0.228 |
|  | Error | 1.8E-4 | 20 |  |  |
|  | Total |  | 23 |  |  |
| Pb | Time : BA | 0.001 | 1 | 0.190 | 0.739 |
|  | Location: CI | 0.006 | 1 | 1.270 | 0.462 |
|  | Interaction: BAxCI | 0.005 | 1 | 2.212 | 0.153 |
|  | Error | 0.041 | 20 |  |  |
|  | Total |  | 23 |  |  |

Data from GT were used as the impact signature and data from FMA were used as the reference (control) signature. BA = before-after, CI=control-impact.
