# Supplementary material for: Is Exposure to Macondo Oil Reflected in the Otolith Chemistry of Marsh-Resident Fish?
Source: PLoS One. 2016 Sep 28;11(9):e0162699. doi: 10.1371/journal.pone.0162699 (PMC5040417; doi:10.1371/journal.pone.0162699)
Supplement: S4 Table — Data from GT were used as the impact signature and data from FMA were used as the reference (control) signature. BA = before-after, CI = control-impact. (DOCX) [file pone.0162699.s004.docx]

**S4 Table. ANOVA table for two-factor BACI design, pre- vs. post-oil comparison**.

| **Element** | **Source** | **Sum of Squares (SS)** | **df** | **F** | ***p*** |
| --- | --- | --- | --- | --- | --- |
| V | Time : BA | 2.9E-5 | 1 | 0.139 | 0.772 |
|  | Location: CI | 0.001 | 1 | 5.812 | 0.250 |
|  | Interaction: BAxCI | 2.1E-4 | 1 | 1.608 | 0.219 |
|  | Error | 0.003 | 20 |  |  |
|  | Total |  | 23 |  |  |
| Mn | Time : BA | 0.033 | 1 | 3.055 | 0.331 |
|  | Location: CI | 0.012 | 1 | 1.095 | 0.486 |
|  | Interaction: BAxCI | 0.011 | 1 | 0.338 | 0.567 |
|  | Error | 0.633 | 20 |  |  |
|  | Total |  | 23 |  |  |
| Ni | Time : BA | 0.030 | 1 | 0.501 | 0.608 |
|  | Location: CI | 0.077 | 1 | 1.294 | 0.459 |
|  | Interaction: BAxCI | 0.060 | 1 | 1.506 | 0.234 |
|  | Error | 0.790 | 20 |  |  |
|  | Total |  | 23 |  |  |
| Cu | Time : BA | 0.030 | 1 | 3.818 | 0.301 |
|  | Location: CI | 0.019 | 1 | 2.383 | 0.366 |
|  | Interaction: BAxCI | 0.008 | 1 | 1.781 | 0.197 |
|  | Error | 0.089 | 20 |  |  |
|  | Total |  | 23 |  |  |
| Sr | Time : BA | 0.036 | 1 | 93.498 | 0.066 |
|  | Location: CI | 0.021 | 1 | 54.283 | 0.086 |
|  | Interaction: BAxCI | 3.9E-4 | 1 | 0.297 | 0.592 |
|  | Error | 0.026 | 20 |  |  |
|  | Total |  | 23 |  |  |
| Ba | Time : BA | 1.7E-4 | 1 | 2.069 | 0.387 |
|  | Location: CI | 8.3E-5 | 1 | 1.014 | 0.498 |
|  | Interaction: BAxCI | 8.2E-5 | 1 | 18.375 | <0.001 |
|  | Error | 8.9E-5 | 20 |  |  |
|  | Total |  | 23 |  |  |
| Pb | Time : BA | 0.021 | 1 | 1.027 | 0.496 |
|  | Location: CI | 0.023 | 1 | 1.121 | 0.482 |
|  | Interaction: BAxCI | 0.021 | 1 | 3.578 | 0.073 |
|  | Error | 0.116 | 20 |  |  |
|  | Total |  | 23 |  |  |

Data from GT were used as the impact signature and data from FMA were used as the reference (control) signature. BA = before-after, CI=control-impact.
